# The triglyceride-glucose index associated with reduced risk of liver metastasis in pancreatic cancer

**DOI:** 10.3389/fendo.2025.1592788

**Published:** 2025-07-18

**Authors:** Taijun Yi, Zejin Lin, Ziyan Mai, Yongling Liang, Chengrui Zhong, Xingyu Li, Wandi Wang, Xiaoyue Huang, Zeyu Lin, Yunle Wan, Guolin Li

**Affiliations:** ^1^ Department of General Surgery (Hepatobiliary, Pancreatic and Splenic Surgery), The Sixth Affiliated Hospital, Sun Yat-sen University, Guangzhou, China; ^2^ Biomedical Innovation Center, The Sixth Affiliated Hospital, Sun Yat-sen University, Guangzhou, China

**Keywords:** TyG index, pancreatic cancer, liver metastasis, tumor pathology, prediction model

## Abstract

**Background:**

The triglyceride-glucose (TyG) index has emerged as a reliable surrogate marker for insulin resistance and is associated with multiple malignancies. However, its role in pancreatic cancer liver metastasis (PCLM) remains unclear. This study aimed to investigate the relationship between TyG index and PCLM and evaluate its predictive value for PCLM.

**Methods:**

This study enrolled 172 patients diagnosed with pancreatic cancer at Sixth Affiliated Hospital of Sun Yat-sen University between 2021 and 2024. Both cross-sectional and longitudinal analyses were employed. Logistic regression, propensity score matching (PSM) and subgroup analysis were utilized to assess the relationship between TyG index and PCLM, and a predictive model was constructed. Kaplan-Meier curves and cox proportional hazards regression analysis were conducted to assess the impact on liver metastasis. LASSO regression and Firth regression were conducted to avoid over-fitting issue. Restricted cubic splines (RCS) were applied to explore the nonlinear relationship.

**Results:**

A significant inverse association was observed between TyG index level and PCLM incidence. Both multivariate logistic and cox regression suggested that a lower TyG index is associated with an increased risk of PCLM. A nomogram model was established and possessed a moderate degree of predictive accuracy (AUC = 0.75, 95% CI = 0.67-0.82). Notably, similar conclusions were reached in the subgroup of pancreatic ductal adenocarcinoma.

**Conclusion:**

Comprehensive analysis suggest that higher TyG index level is associated with reduced risk for PCLM, offering significant guidance for the prediction and early intervention of PCLM.

## 1 Introduction

Pancreatic cancer is among the most prevalent malignancies within the digestive system. Its incidence and mortality rates are on the relentless rise, while its survival rate remains a poor rate of less than 10% (1). The early stages of pancreatic cancer often elude detection due to a lack of significant symptoms. By the time of diagnosis, the disease frequently presents with metastasis, most notably to the liver, which accounts for 37% to 41.9% of initial diagnosis cases (2, 3). More critically, patients with liver metastasis face a significantly poorer prognosis compared to those with other sites (4). Therefore, understanding the biological behavior and clinical characteristics of pancreatic cancer liver metastasis (PCLM) is of great importance for its diagnosis and treatment.

Insulin resistance (IR) is a hallmark of metabolic syndrome (5), intimately linked to a variety of diseases. For diagnosing IR, the hyperinsulinemic-euglycemic clamp method is the gold standard (6). However, this method is cumbersome, costly, and technically demanding, which limit its clinical application. Conversely, the triglyceride-glucose index (TyG index) has emerged as a practical and effective measure of IR (7) and identified as a significant risk factor for a spectrum of diseases, including cardiovascular and cerebrovascular diseases (8, 9), renal diseases (10), etc. Notably, studies have begun to explore the relationship between TyG index and tumors, including colorectal, lung, breast, and prostate cancers (11–16), yielding inconsistent conclusions. TyG index appears to be closely intertwined to pancreatic cancer, as both are associated with dysregulations in glucose and lipid metabolism (16, 17), systemic inflammation (18, 19) and immune responses (20), etc. However, how does the TyG index fluctuate during the progression of PCLM, and what are its effects on PCLM? These questions are of significant importance.

In this study, we investigated the relationship between TyG index and PCLM and evaluated its predictive value for PCLM.

## 2 Material and method

**Figure 1:**
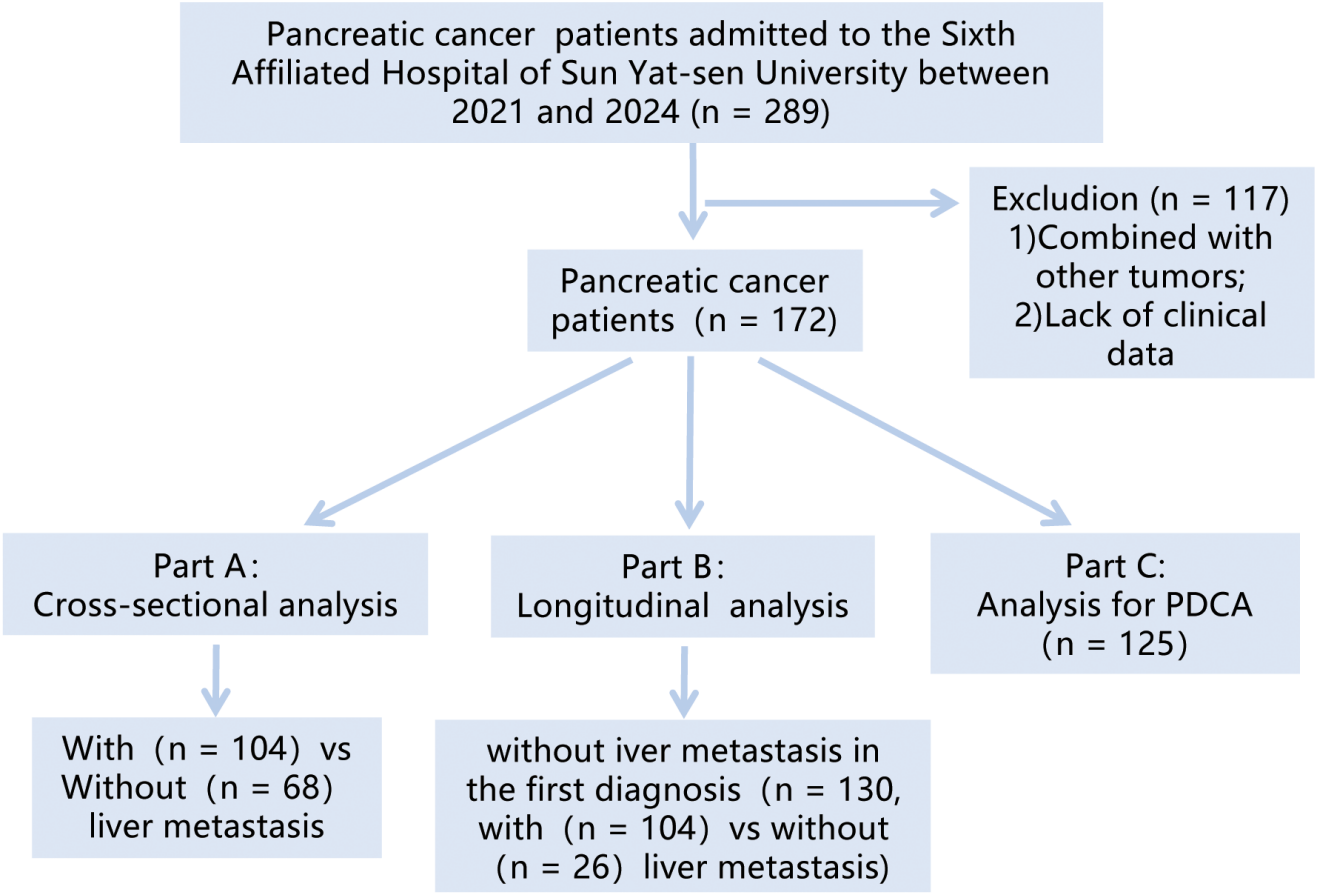
Patients flow diagram.

**Figure 1** depicted the flowchart of the patient cohort. This study retrospectively analyzed patients diagnosed with “pancreatic tumor” at Sixth Affiliated Hospital of Sun Yat-sen University from 2021 to 2024. The research is analyzed from two perspectives. Firstly, a cross-sectional study was conducted to analyze the correlation between the TyG index and PCLM. Subsequently, a longitudinal study was conducted to select patients without liver metastasis at initial diagnosis, aiming to explore the predictive ability of the TyG index for the occurrence of PCLM.

The inclusion criteria were: 1) patients who were first diagnosed at our hospital; 2) pathologically confirmed pancreatic tumors; 3) patients with complete fasting blood glucose (FBG), triglyceride (TG) and essential basic information. The exclusion criteria were: 1) patients with other tumors; 2) patients with missing important data such as FBG and TG. After screening, a total of 172 patients were included in this study.

The main data collected in this study included: 1) basic information: gender, age, BMI, situation of hypertension and diabetes; 2) tumor information: CA199 (U/ml), CA125 (U/ml), CEA (ng/ml), pathological type, T and N stage, liver metastasis situation, follow-up time; 3) test indicators: Fasting blood glucose (FBG, mg/dl), triglyceride (TG, mg/dl), total cholesterol (TC, mmol/l), high-density lipoprotein (HDL, mmol/l), low-density lipoprotein (LDL, mmol/l), albumin (Alb, g/l), total bilirubin (Tbib, umol/l), C-reactive protein (CRP, mg/l), and activated partial thromboplastin time (APTT, s). The blood test results were obtained at the initial diagnosis, 6 a.m. and on an empty stomach > 8h. The liver metastasis was comprehensively evaluated by CT and MR. We subsequently calculated TyG index and Albumin-Bilirubin Index (ALBI) (21) using established formulas:


$$\text{TyG}=\text{ln }\left[\text{TG}*\text{FBG}/2\right]$$

$$\text{ALBI}=\text{lg}\left(\text{Tbib}*0.66-0.085*\text{Alb}\right)$$

The collected data were processed as follows. In the collected data, there were missing values for tumor markers (CA199, CA125, CEA), CRP, and APTT, which were filled by means. Men with values higher than 8.81 and women with values higher than 8.73 were classified as high TyG index level, indicating a state of insulin resistance. Age was grouped with 65 years as the cutoff, and CA199, CA125, and CEA were respectively grouped with critical values of 37 U/ml, 35 U/ml, and 5 ng/ml. Pathological types were divided into three categories: pancreatic ductal adenocarcinoma (PDAC), ampulla cancer (AC), and others (other tumors in pancreatic head region, including papillary tumors and adenocarcinomas of the duodenum, bile duct cancer, Intraductal papillary mucinous neoplasm (IPMN), and neuroendocrine tumors). The final follow-up time for patients was recorded as the time of their last hospital treatment.

Statistical analysis was conducted using R.4.2. Given the skewed distribution of continuous variable data in this study, median and interquartile range were used for description, and Mann-Whitney test were performed for analysis. Categorical variables were described using percentages and analyzed using chi-square tests and Fisher’s exact tests. This study combined cross-sectional and longitudinal analyses, applying logistic regression and Cox regression to jointly assess risk factors. Propensity score matching (PSM) was used to reduce group differences, LASSO regression and Firth regression were conducted to avoid over-fitting issue. And subgroup analysis was used to assess the variability of independent variables in subgroups. We also established a nomogram to assess the risk of metastasis and used the receiver operating curve (ROC) to evaluate predictive efficacy. Kaplan-Meier (K-M) curves were used to assess the impact, and restricted cubic splines (RCS) curves were also used to explore nonlinear relationships. P <0.05 was considered statistically significant. Adobe illustrator and Graphpad prism were used for image processing.

We confirm that our study was performed in accordance with relevant guidelines and regulations and applied for exemption from the informed consent form. This study protocol was reviewed and approved by Ethics Committee of the Sixth Affiliated Hospital, Sun Yat-sen University (2025ZSLYEC-049).

## 3 Results

### 3.1 Liver metastasis group vs non-liver metastasis group

#### 3.1.1 Difference analysis and logistic regression

A cohort of 172 pancreatic cancer patients were enrolled in this study, with 104 (60.47%) exhibiting no liver metastasis and 68 (39.53%) presenting with liver metastasis at initial diagnosis or during subsequent follow-up. Regardless of the analytical approach—numerical or categorical—patients with liver metastasis consistently demonstrated lower TyG index levels compared to the non-metastatic counterparts. Univariate analysis (OR = 0.4, 95% CI = 0.22~0.75, p = 0.005) and multivariate analysis (OR = 0.28, 95% CI = 0.132~0.61, p = 0.001) both indicated that a lower TyG index is associated with an increased risk of liver metastasis. Additionally, results highlighted age ≥65 years was related to a lower risk of liver metastasis, while diabetes and N emerged as risk factors. C-reactive protein (CRP) did not show statistical significance (**Tables 1**, **2**).

**Table 1:** Baseline characteristics and differences analysis for patients before PSM.

Variables	Total (n = 172)	non-LM (n = 104)	LM (n = 68)	Statistic	*P*
TyG index, M (Q_1_, Q_3_)	8.80 (8.45, 9.37)	8.92 (8.54, 9.39)	8.55 (8.27, 9.17)	Z=-2.74	**0.006**
TyG level, n(%)				χ²=8.22	0.004
low	83 (48.26)	41 (39.42)	42 (61.76)		
high	89 (51.74)	63 (60.58)	26 (38.24)		
Sex, n(%)				χ²=0.37	0.541
male	106 (61.63)	66 (63.46)	40 (58.82)		
female	66 (38.37)	38 (36.54)	28 (41.18)		
Age, n(%)				χ²=3.88	0.049
<65	98 (56.98)	53 (50.96)	45 (66.18)		
≥65	74 (43.02)	51 (49.04)	23 (33.82)		
BMI, M (Q_1_, Q_3_)	21.27 (19.03, 23.31)	21.45 (19.48, 23.60)	20.93 (18.68, 22.95)	Z=-0.86	0.387
Hypertension, n(%)				χ²=0.00	0.985
no	139 (80.81)	84 (80.77)	55 (80.88)		
yes	33 (19.19)	20 (19.23)	13 (19.12)		
Diabetes, n(%)				χ²=0.46	0.5
no	99 (57.56)	62 (59.62)	37 (54.41)		
yes	73 (42.44)	42 (40.38)	31 (45.59)		
Pathologytype, n(%)				χ²=7.27	0.026
PDAC	125 (72.67)	68 (65.38)	57 (83.82)		
AC	18 (10.47)	13 (12.50)	5 (7.35)		
Others	29 (16.86)	23 (22.12)	6 (8.82)		
T, n(%)				χ²=11.43	0.01
1	17 (9.88)	13 (12.50)	4 (5.88)		
2	32 (18.60)	23 (22.12)	9 (13.24)		
3	51 (29.65)	35 (33.65)	16 (23.53)		
4	72 (41.86)	33 (31.73)	39 (57.35)		
N, n(%)				χ²=14.16	<.001
0	85 (49.42)	63 (60.58)	22 (32.35)		
1	59 (34.30)	30 (28.85)	29 (42.65)		
2	28 (16.28)	11 (10.58)	17 (25.00)		
CA199, n(%)				χ²=1.60	0.206
<37	63 (36.63)	42 (40.38)	21 (30.88)		
≥37	109 (63.37)	62 (59.62)	47 (69.12)		
CA125, n(%)				χ²=6.84	0.009
<35	116 (67.44)	78 (75.00)	38 (55.88)		
≥35	56 (32.56)	26 (25.00)	30 (44.12)		
CEA, n(%)				χ²=0.47	0.495
<5	114 (66.28)	71 (68.27)	43 (63.24)		
≥5	58 (33.72)	33 (31.73)	25 (36.76)		
TC, M (Q_1_, Q_3_)	4.45 (3.69, 5.26)	4.54 (3.79, 5.39)	4.34 (3.52, 5.19)	Z=-0.95	0.34
HDL, M (Q_1_, Q_3_)	1.07 (0.83, 1.35)	1.04 (0.81, 1.36)	1.12 (0.91, 1.29)	Z=-1.03	0.303
LDL, M (Q_1_, Q_3_)	3.12 (2.51, 3.77)	3.11 (2.66, 3.77)	3.16 (2.42, 3.77)	Z=-0.50	0.616
ALBI, M (Q_1_, Q_3_)	-2.67 (-2.92, -2.29)	-2.59 (-2.93, -2.26)	-2.71 (-2.92, -2.37)	Z=-0.94	0.347
CRP, M (Q_1_, Q_3_)	8.63 (2.76, 20.26)	7.10 (2.73, 20.26)	14.83 (3.03, 20.26)	Z=-1.35	0.176
APTT, M (Q_1_, Q_3_)	30.65 (28.25, 32.92)	30.85 (28.87, 33.02)	30.30 (27.60, 32.50)	Z=-1.42	0.155

Z: Mann-Whitney test, χ²: Chi-square test.

M, Median; Q_1_, 1st Quartile; Q_3_, 3st Quartile.

non-LM, patients without liver metastasis.

LM, patients with liver metastasis.

The bold values indicate p < 0.05.

**Table 2:** Univariate and multivariate logistic regression for patients before PSM.

Variables	Univariate					Multivariate				
	β	S.E	Z	P	OR (95%CI)	β	S.E	Z	*P*	OR (95%CI)
TyG level										
low					1.00 (Reference)					1.00 (Reference)
high	-0.91	0.32	-2.84	**0.005**	0.40 (0.22 ~ 0.75)	-1.27	0.4	-3.2	**0.001**	0.28 (0.13 ~ 0.61)
Sex										
male					1.00 (Reference)					
female	0.2	0.32	0.61	0.541	1.22 (0.65 ~ 2.27)					
Age										
<65					1.00 (Reference)					1.00 (Reference)
≥65	-0.63	0.32	-1.96	0.05	0.53 (0.28 ~ 0.99)	-0.81	0.36	-2.23	**0.025**	0.44 (0.22 ~ 0.91)
BMI	-0.05	0.05	-1.06	0.289	0.95 (0.87 ~ 1.04)					
Hypertension										
no					1.00 (Reference)					
yes	-0.01	0.4	-0.02	0.985	0.99 (0.46 ~ 2.16)					
Diabetes										
no					1.00 (Reference)					1.00 (Reference)
yes	0.21	0.31	0.67	0.5	1.24 (0.67 ~ 2.29)	0.9	0.4	2.24	**0.025**	2.46 (1.12 ~ 5.39)
Pathologytype										
PDAC					1.00 (Reference)					
AC	-0.78	0.56	-1.4	0.161	0.46 (0.15 ~ 1.36)					
Others	-1.17	0.49	-2.37	**0.018**	0.31 (0.12 ~ 0.82)					
T										
1					1.00 (Reference)					
2	0.24	0.69	0.35	0.729	1.27 (0.33 ~ 4.96)					
3	0.4	0.65	0.61	0.54	1.49 (0.42 ~ 5.28)					
4	1.35	0.62	2.17	**0.03**	3.84 (1.14 ~ 12.92)					
N										
0					1.00 (Reference)					1.00 (Reference)
1	1.02	0.36	2.83	**0.005**	2.77 (1.37 ~ 5.60)	0.93	0.39	2.37	**0.018**	2.53 (1.17 ~ 5.46)
2	1.49	0.46	3.24	**0.001**	4.43 (1.80 ~ 10.89)	1.61	0.49	3.28	**0.001**	4.98 (1.91 ~ 12.99)
CA199										
<37					1.00 (Reference)					
≥37	0.42	0.33	1.26	0.207	1.52 (0.79 ~ 2.89)					
CA125										
<35					1.00 (Reference)					
≥35	0.86	0.33	2.59	**0.01**	2.37 (1.23 ~ 4.55)					
CEA										
<5					1.00 (Reference)					
≥5	0.22	0.33	0.68	0.495	1.25 (0.66 ~ 2.38)					
TC	-0.12	0.09	-1.33	0.182	0.89 (0.75 ~ 1.06)					
HDL	0.05	0.12	0.37	0.712	1.05 (0.82 ~ 1.33)					
LDL	-0.07	0.12	-0.55	0.581	0.93 (0.73 ~ 1.19)					
ALBI	-0.16	0.24	-0.65	0.518	0.86 (0.53 ~ 1.37)					
CRP	0.01	0	1.53	0.127	1.01 (1.00 ~ 1.02)	0.01	0	1.58	0.114	1.01 (1.00 ~ 1.02)
APTT	-0.06	0.04	-1.4	0.16	0.94 (0.87 ~ 1.02)					

OR, Odds Ratio; CI, Confidence Interval. The bold values indicate p < 0.05.

#### 3.1.2 PSM

Accounting for patients’ fundamental conditions—such as gender, age, hypertension, and diabetes, as well as other lipid-related indicators (TC, HDL, and LDL), liver function indicators (ALBI and APTT), and inflammatory markers (CRP), PSM was performed. After PSM, the analysis included 70 patients: 41 without liver metastasis and 29 with liver metastasis. The group with liver metastasis exhibited significantly lower TyG index levels (p = 0.009), and both univariate analysis (OR = 0.34, 95% CI = 0.13-0.91, p = 0.031) and multivariate analysis (OR = 0.17, 95% CI = 0.04-0.68, p = 0.012) reinforced the notion that a lower TyG index is associated with liver metastasis. (**Supplementary Tables 1**, **2**, **Supplementary Figures 1**, **2**).

#### 3.1.3 Subgroup analysis

**Figure 2:**
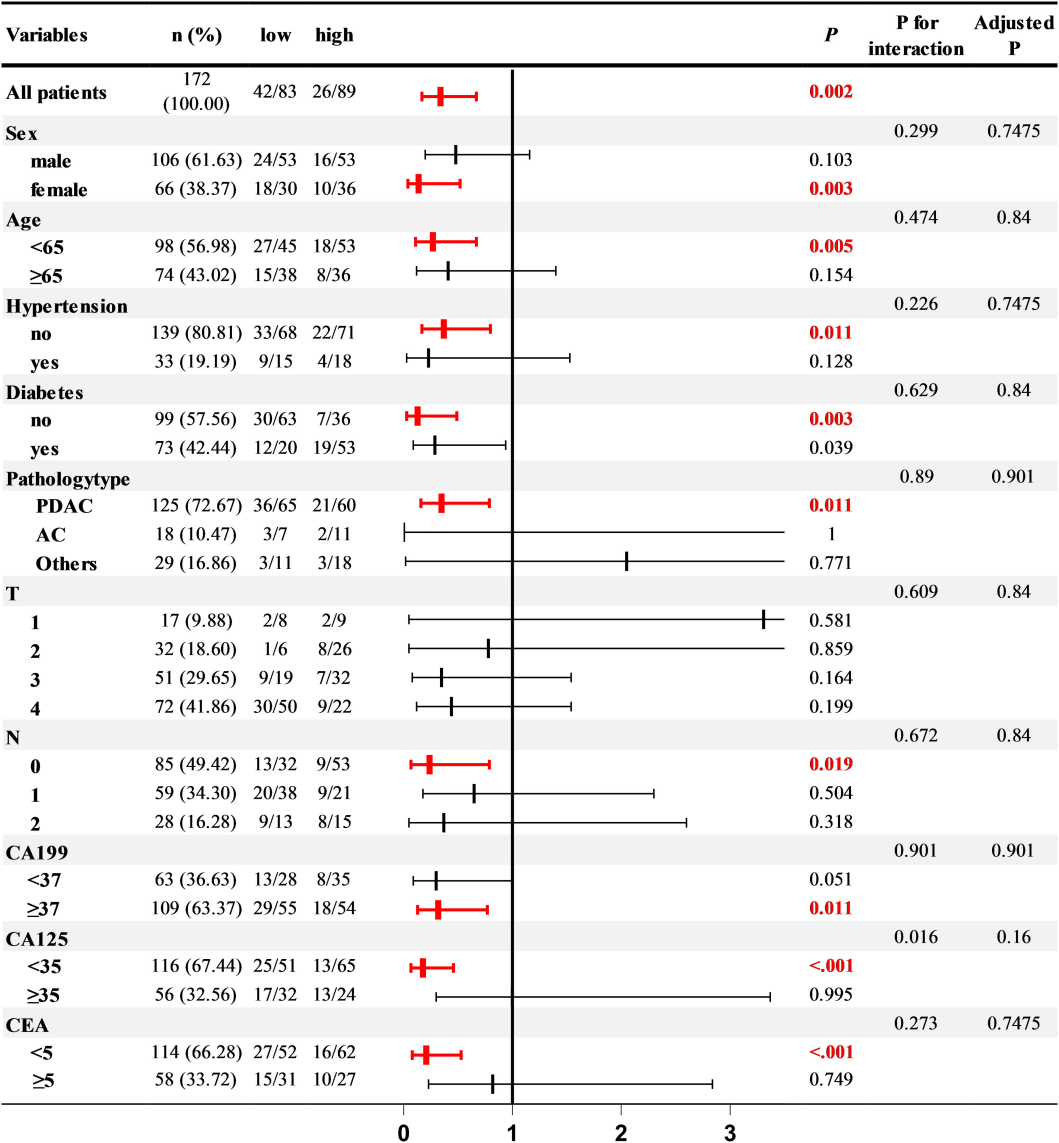
Subgroup analysis.

To delve deeper into the relationship between PCLM and the TyG index under various conditions, we conducted a subgroup analysis. After adjusting for lipid-related indicators (TC, HDL, LDL), liver function indicators (ALBI, APTT), and inflammatory markers (CRP), the correlation between the TyG index and PCLM remained significant across most subgroups. Notably, the association was more pronounced in women, individuals under 65 years of age, those without hypertension, and in patients with or without diabetes, PDAC, N0, CA199 ≥37, and normal CA125 and CEA levels (**Figure 2**).

#### 3.1.4 Clinical prediction model

Utilizing variables from the multivariable regression analysis conducted prior to PSM, we developed a nomogram to predict the likelihood of PCLM occurrence. The nomogram demonstrated moderate predictive accuracy, with an area under the curve (AUC) of 0.75 (95% confidence interval [CI]: 0.67-0.82) (**Supplementary Figures 3**-**6**).

### 3.2 Analysis on patients without liver metastasis at initial diagnosis

#### 3.2.1 Difference analysis and K-M curve

Among the 172 patients in this study, 130 were free of liver metastasis at initial diagnosis and were subject to further investigation. Over a median follow-up period of 190 days (range, 3–1237 days), 26 (20%) developed liver metastasis. Consistent with findings above, TyG index levels varied significantly between the two groups, whether considered as numerical or categorical data. The K-M curve revealed that patients with higher TyG index levels had a reduced likelihood of developing liver metastasis (log-rank P = 0.02, HR(95% CI) = 0.285(0.125~0.650)) (**Supplementary Table 3**, **Supplementary Figures 7**-**9**).

#### 3.2.2 Multimodal and multifactorial cox regression

Subsequently, we performed Cox regression analysis. The multimodal Cox regression indicated that, after sequentially accounting for all covariates—including gender, age, and BMI—a higher TyG index level continued to exhibit a protective effect against PCLM. The multivariate Cox regression analysis corroborated this finding (HR = 0.41, 95% CI = 0.17-0.99) and further identified N2 as a risk factor for PCLM (HR = 4.64, 95% CI = 1.55-13.89). (**Supplementary Tables 4**, **5**). We conducted LASSO regression and Firth regression, and selected three variables for cox regression based on the results of LASSO regression and Firth regression, all of which indicated the same conclusion (**Supplementary Table 6**). The prior power calculation demonstrated 80% power to detect HR=0.4 (**Supplementary Table 7**).

#### 3.2.3 RCS curve

In light of the potential nonlinear relationship between the TyG index and liver metastasis, we plotted the RCS curve. Both before and after adjusting for all covariates, the RCS curves consistently indicated a nonlinear association between the TyG index and liver metastasis. (**Supplementary Figures 10**, **11**)

### 3.3 PDAC

As the most prevalent pathological type of pancreatic cancer, PDAC was subjected to further analysis in this study. We analyzed all PDAC patients (n = 125, with 68 having liver metastasis and 57 without) for difference analysis and logistic regression analysis. Additionally, PDAC patients without liver metastasis at initial diagnosis (n = 87, with 68 developing liver metastasis and 19 remaining free) were analyzed using Cox regression and K-M curve plotting. Aligning with our previous findings, the metastatic group exhibited significantly lower TyG index levels compared to the non-metastatic group in both scenarios (**Supplementary Tables 8.1**, **9.1**). Both logistic and Cox regression analyses suggested that a low TyG index level is a risk factor for PCLM (**Supplementary Tables 8.2**, **9.2**). The K-M curve indicated that patients with lower TyG index levels had a higher likelihood of developing liver metastasis [log-rank P = 0.004, HR (95% CI) = 0.255(0.094-0.689)] (**Supplementary Figures 12**-**14**).

## 4 Discussion

The relationship between TyG index and tumor liver metastasis hasn’t been extensively studied. Our study, employing both cross-sectional and longitudinal analyses, revealed a correlation between higher TyG index level and reduced risk of PCLM.

Given the potential correlation between IR and tumorigenesis, the TyG index, a novel IR indicator, has garnered attention for its potential impact on tumors. However, previous studies have presented conflicting conclusions. While the majority suggest that TyG index is a high-risk factor for tumor occurrence and progression, including breast cancer (22), colorectal cancer (11, 16) and lung cancer (13), some studies indicate no association or reverse conclusion in lung (15), prostate cancer (14), and female reproductive system tumors (16). There are few studies on the correlation between TyG index and PCLM. Although a study suggested that TyG index is related to the risk of pancreatic cancer (16), Song et al. (23) found that TyG index levels in pancreatic cancer with distant metastasis are paradoxically lower compared to early pancreatic cancer. This is the only article on the correlation between TyG index and pancreatic cancer with metastasis, and our study came to similar results as them.

It is well known that IR is associated with high insulin levels, so most studies tend to use this to explain the possible mechanism of TyG index as a cancer risk. For example, insulin can directly activate the PI3K/AKT/MTOR/S6K signaling pathway in tumors (24), and promote the increase of NF-KB to regulate cell proliferation and tumor metastasis (11). And IR is associated with increased levels of IGF-1, which activates the IGF-1 receptor (25) or promotes the production of vascular endothelial growth factor, thereby promoting tumor cell proliferation and angiogenesis.

While our study revealed that TyG index was inversely related to PCLM, which may be explained as follows. Firstly, pancreatic cancer and IR exhibit contrasting characteristics: pancreatic cancer is often marked by reduced insulin secretion (26), while the latter manifests as hyperinsulinemia, indicating a more complex mechanism than other tumors. Song et al. (23) proposed a compensatory mechanism for metabolic or hormonal pathways during PDAC progression. Early-stage pancreatic cancer is characterized by IR and compensatory hyperinsulinemia, whereas advanced pancreatic cancer is characterized by a reduction in IR and insulin secretion to establish a new metabolic equilibrium. Therefore, advanced pancreatic cancer is characterized by a decrease in TyG index levels. Of course, this mechanism may not apply to ampulla cancer or some specific tumors. We also consider that cachexia-induced microenvironment changes may contribute to the observed decrease in the TyG index. While pancreatic cancer is frequently associated with hyperglycemia and cachexia-related hypertriglyceridemia (23, 27), emerging evidence indicates that advanced cachexia may paradoxically enhance gluconeogenesis and lipid catabolism, depleting systemic glucose and triglyceride reserves (28–31). This complex metabolic process may result in a reduced TyG index. Additionally, changes in liver metabolism and microenvironment also affect TyG index level (32, 33). For example, these changes promote the secretion of pro-inflammatory cytokines such as interleukin-6, tumor necrosis factor-a, and interleukin-1, thereby inhibiting the synthesis of triglycerides (34). Therefore, despite some contradictions with previous studies, our findings are plausible.

Several limitations should be acknowledged. Firstly, this study didn’t dynamically monitor the TyG Index, thus failing to assess its changes over the disease process, which could provide more meaningful insights and warrants future investigation. Secondly, the retrospective and single-center nature of this study limits generalizability to other ethnicities, health-care settings, and earlier disease stages. Additionally, the study didn’t account for certain confounding factors that could influence the TyG index, such as patients’ dietary habits, the use of lipid-lowering and hypoglycemic medications, and underlying conditions like cardiovascular diseases, hyperlipidemia and cachexia. Furthermore, while our predictive model demonstrates some predictive value, it necessitates external validation and larger sample sizes for enhancement.

In conclusion, our findings identified that higher TyG index levels are associated with reduced risk for PCLM, highlighting its utility in prognostication and early intervention. These results help to elucidate the metabolic characteristics and disease prediction of PCLM, but the underlying mechanisms still need further exploration.

## Data Availability

The raw data supporting the conclusions of this article will be made available by the authors, without undue reservation.
