# Supplementary material for: The triglyceride-glucose index associated with reduced risk of liver metastasis in pancreatic cancer
Source: Front Endocrinol (Lausanne). 2025 Jul 18;16:1592788. doi: 10.3389/fendo.2025.1592788 (PMC12314753; doi:10.3389/fendo.2025.1592788)
Supplement: Supplementary file 1 [file DataSheet1.pdf]

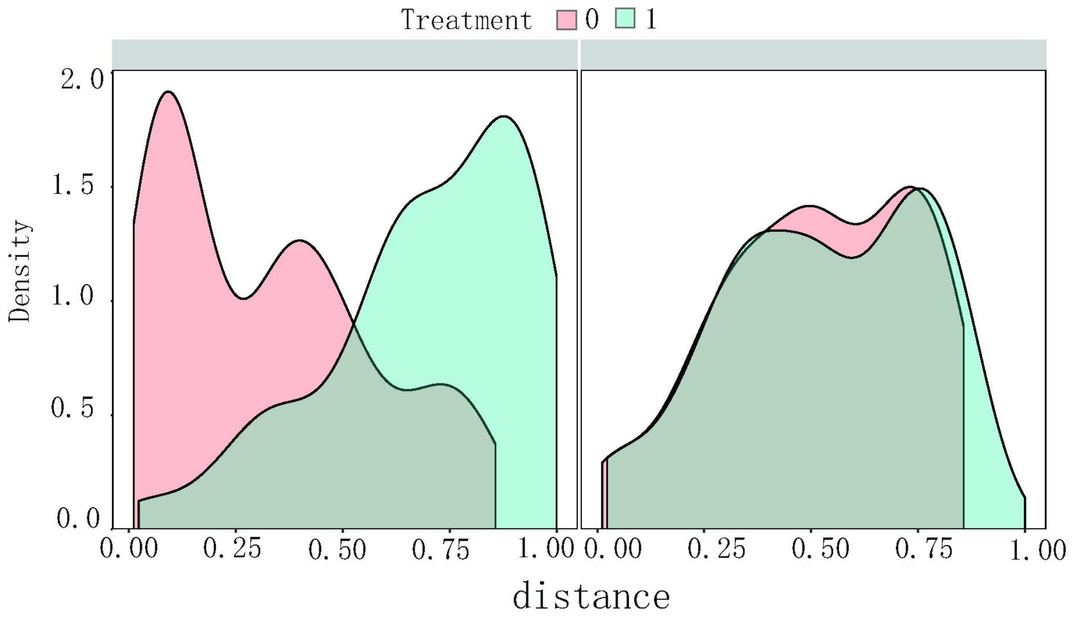

Supplementary Fig 1. probability density analysis diagram of PSM.

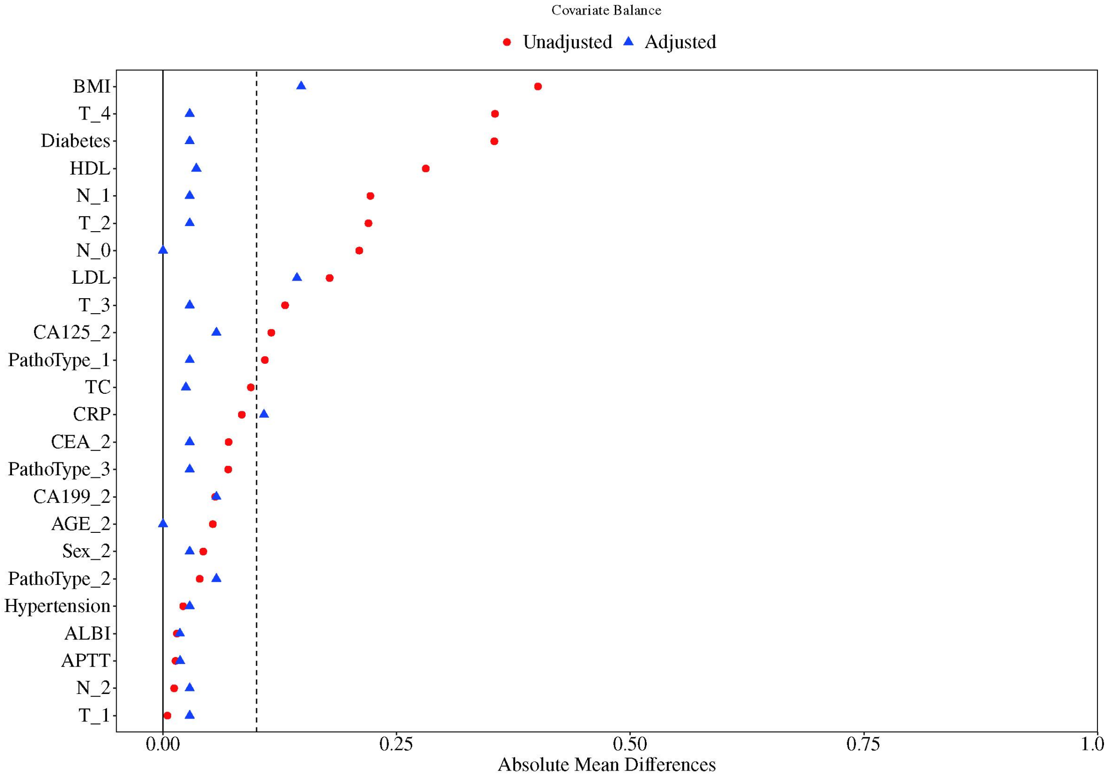

Supplementary Fig 2. SMD analysis diagram of PSM.

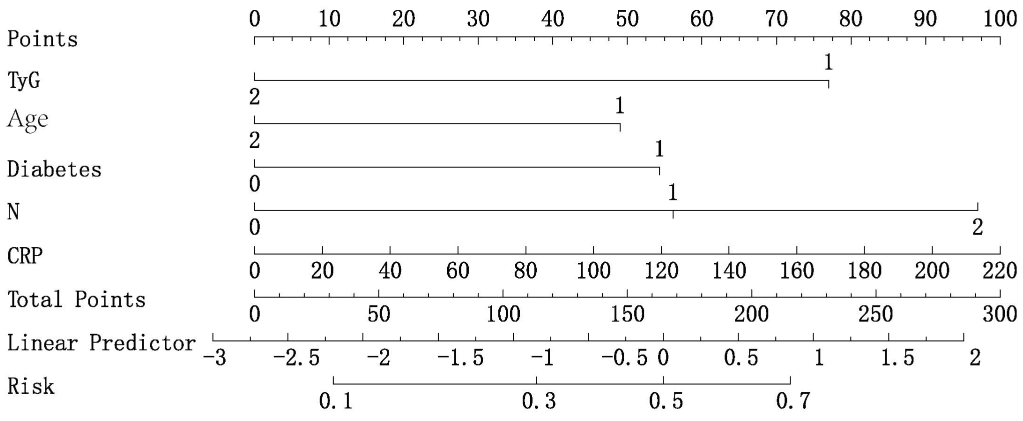

Supplementary Fig 3. nomogram developed by variables from the multivariable regression analysis conducted prior to PSM.

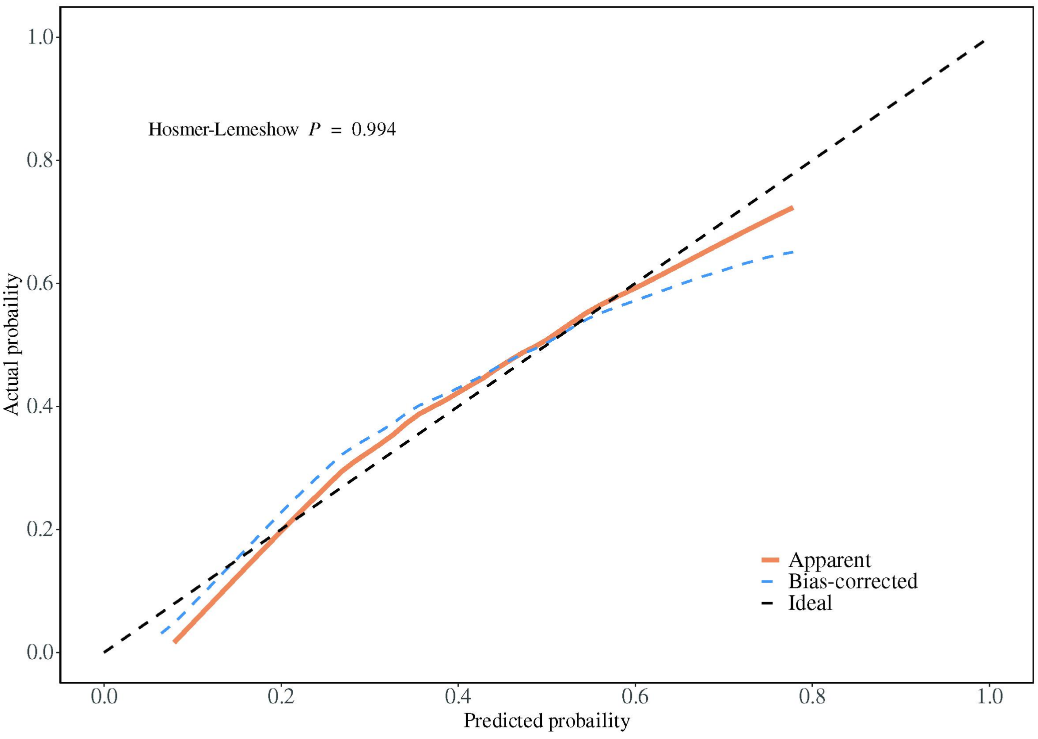

Supplementary Fig 4. Calibration Curve for the nomogram.

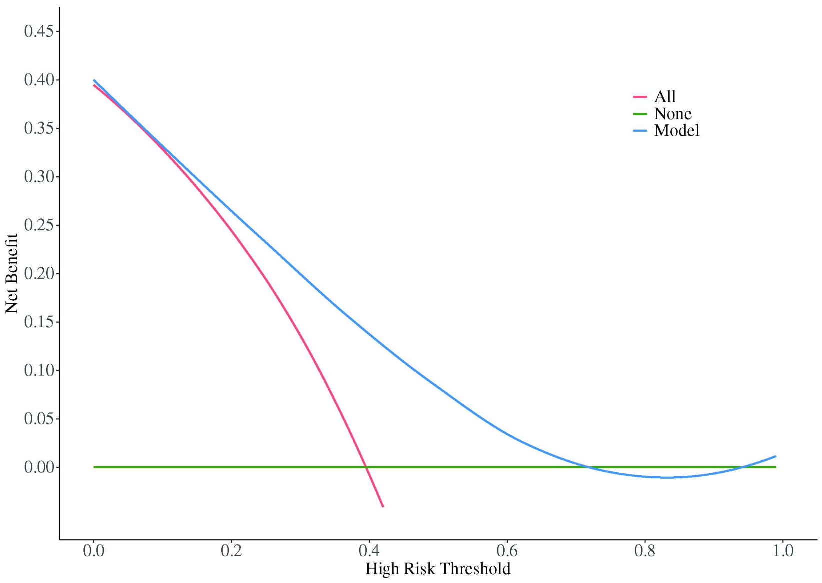

Supplementary Fig 5. DCA curve for the nomogram.

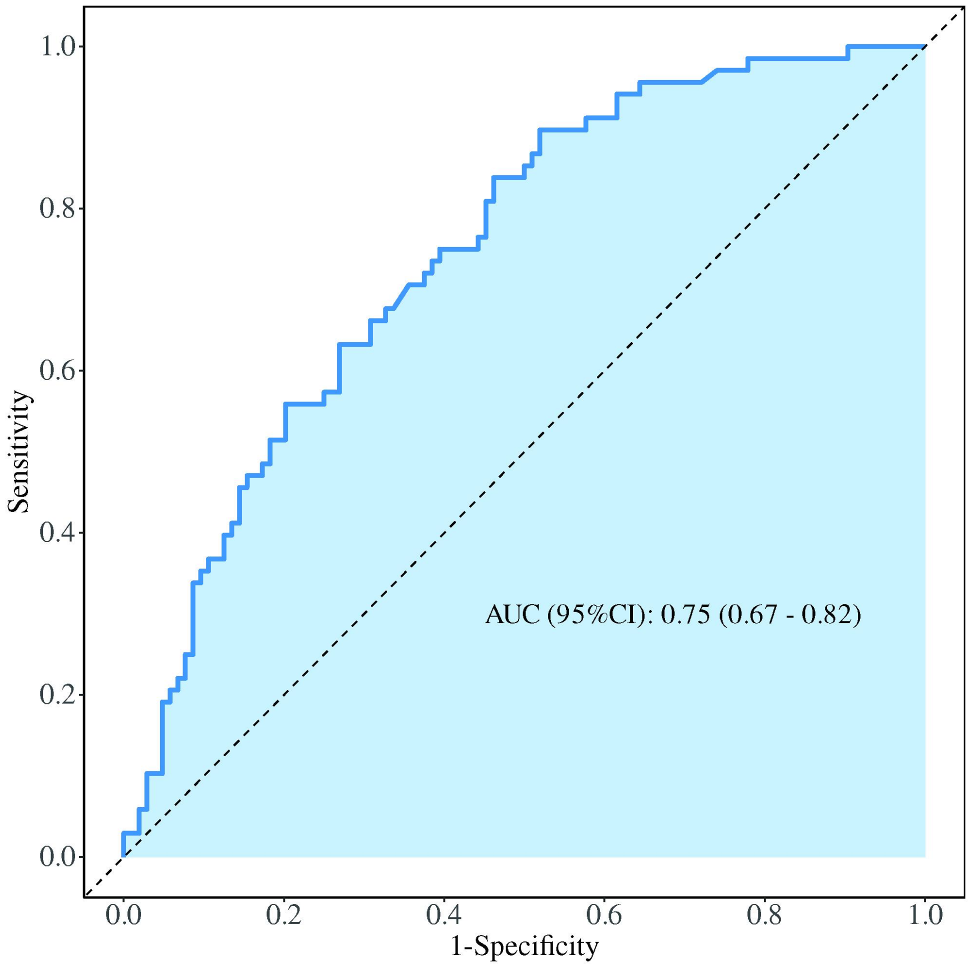

Supplementary Fig 6. the ROC curve for the nomogram, indicating a moderate degree of predictive accuracy (AUC = 0.75, 95% CI = 0.67~0.82) for the model.

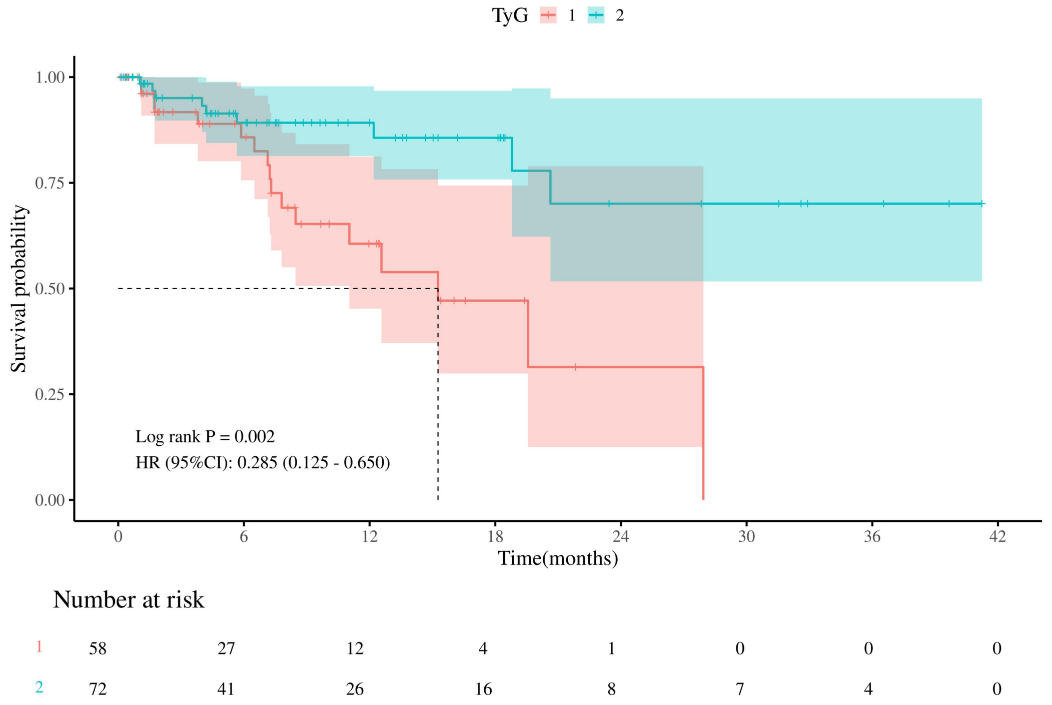

Supplementary Fig 7. the K-M curve for patients without liver metastasis at initial diagnosis.

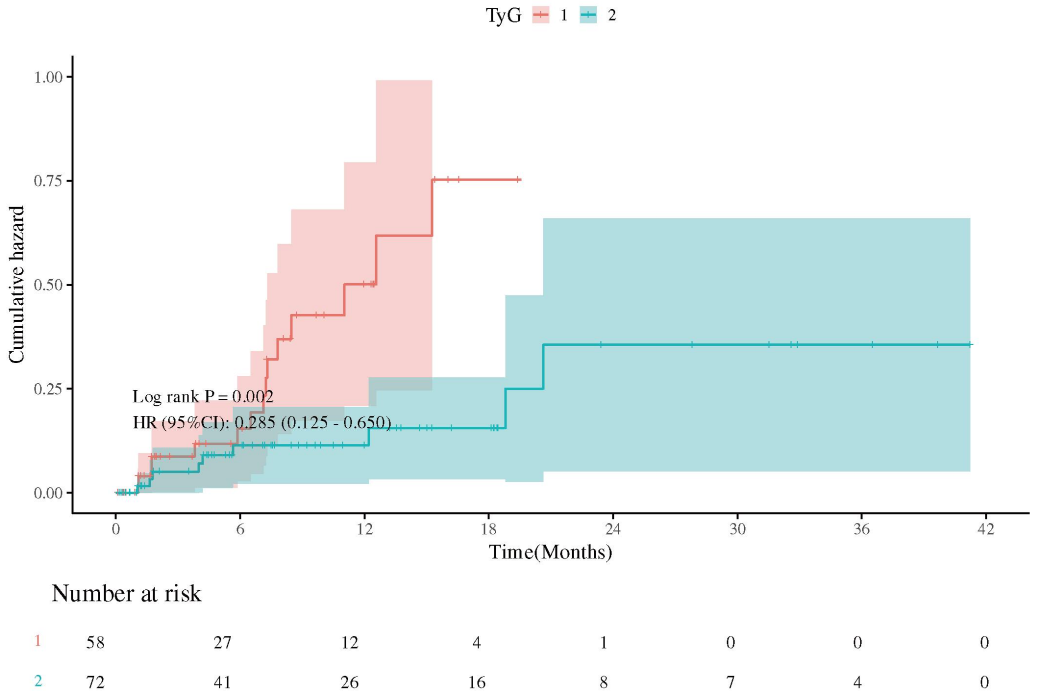

Supplementary Fig 8. the cumulative hazard curve for patients without liver metastasis at initial diagnosis.

Variables	N	Events	Median (95%CI)	Rate/1000 (person-months)	*Logrank P value*
TyG					**0.002**
1	58	17	15.27 (8.47 - NA)	15454.55	
2	72	9	NA (20.63 - NA)	8709.68	

Supplementary Fig 9. the median survival schedule for patients without liver metastasis at initial diagnosis.

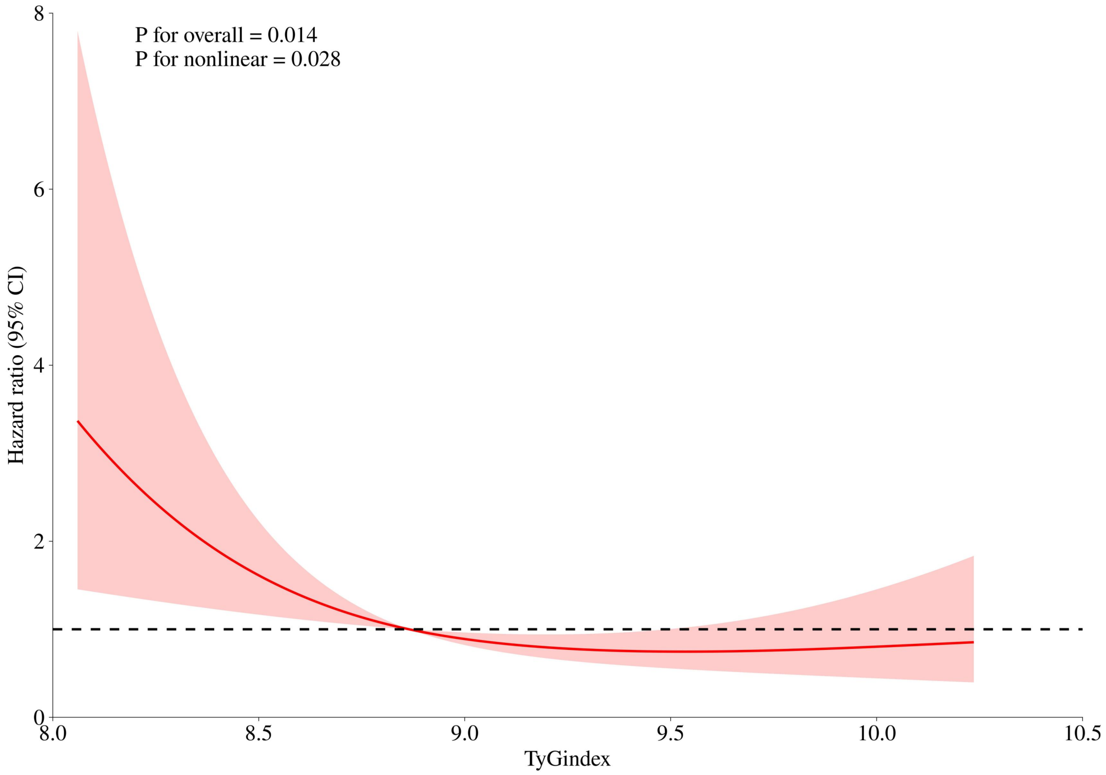

Supplementary Fig 10. RCS curve before adjusting for all covariates.

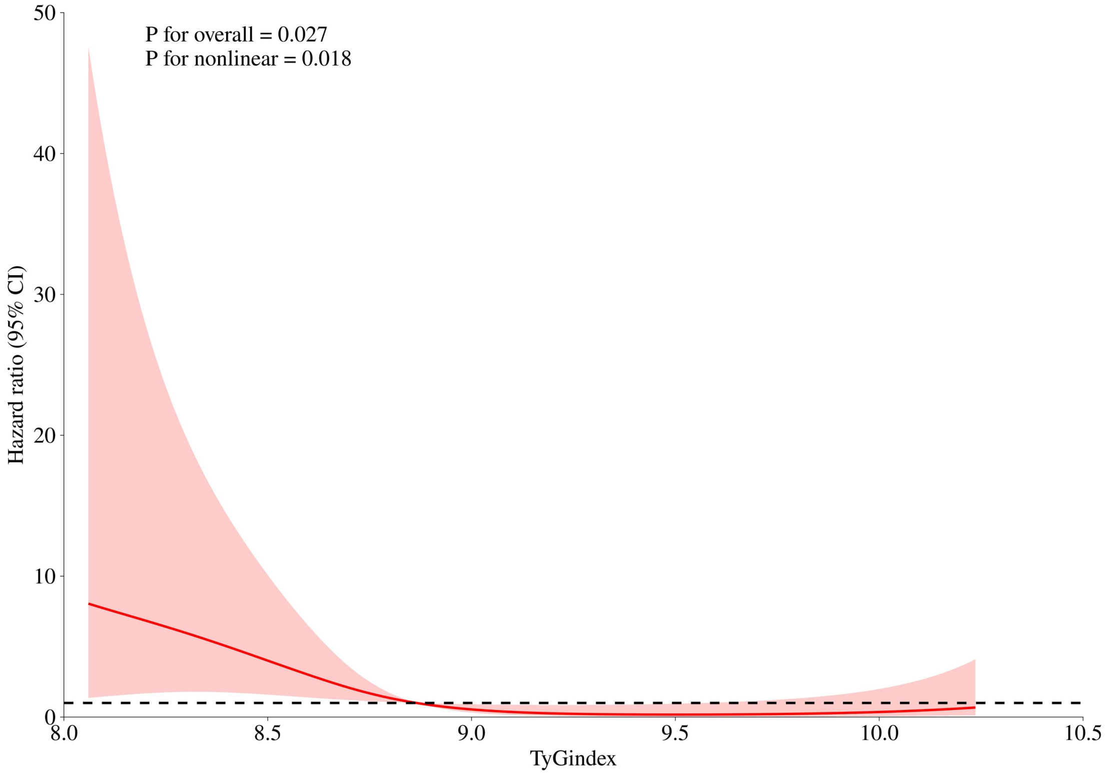

Supplementary Fig 11. RCS curve after adjusting for all covariates.

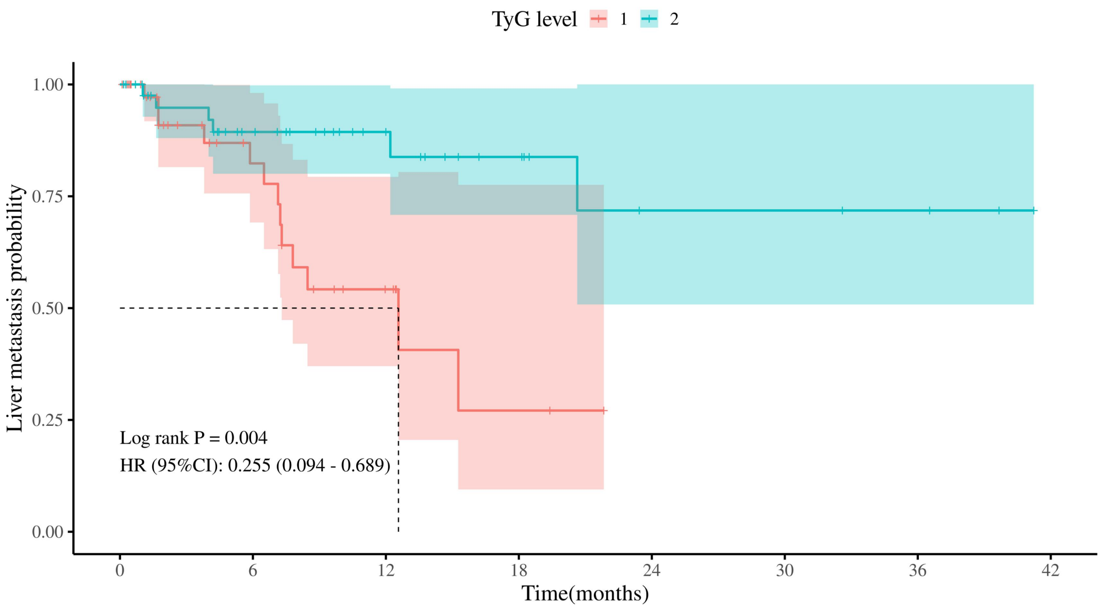

Number at risk

1	42	18	7	2	0	0	0	0
2	45	27	17	10	5	5	4	0

Supplementary Fig 12. the K-M curve for PDAC patients without liver metastasis at initial diagnosis.

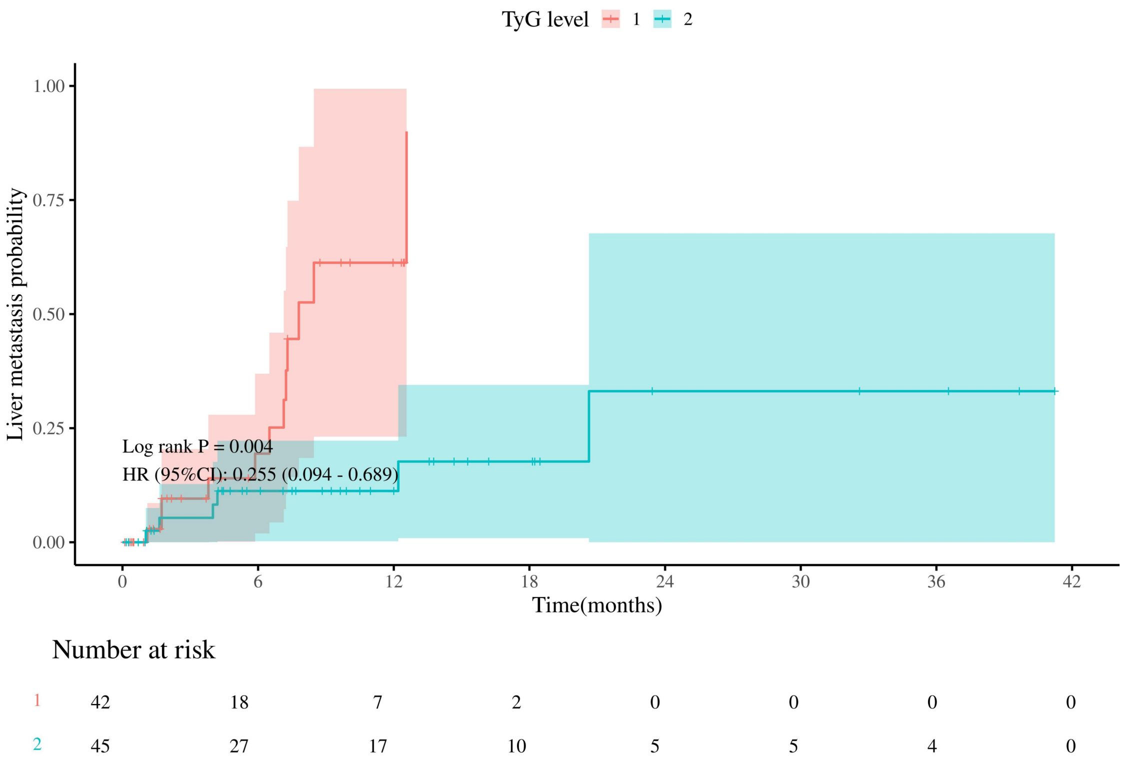

Supplementary Fig 13. the cumulative hazard curve for PDAC patients without liver metastasis at initial diagnosis.

Variables	N	Events	Median (95%CI)	Rate/1000 (person-years)	*Logrank P value*
TyG					**0.004**
1	42	13	12.57 (7.30 - NA)	11818.18	
2	45	6	NA (NA - NA)	5806.45	

Supplementary Fig 14. the median survival schedule for PDAC patients without liver metastasis at initial diagnosis.
